# Elevated CO_2_ Can Worsen Fusarium Head Blight Disease Severity in Wheat but the *Fhb1* QTL Provides Reliable Disease Resistance

**DOI:** 10.3390/plants12203527

**Published:** 2023-10-11

**Authors:** William T. Hay, James A. Anderson, David F. Garvin, Susan P. McCormick, Mark Busman, Martha M. Vaughan

**Affiliations:** 1 USDA, Agricultural Research Service, National Center for Agricultural Utilization Research, Mycotoxin Prevention and Applied Microbiology Research Unit, 1815 N, University Street, Peoria, IL 61604, USA; susan.mccormick@usda.gov (S.P.M.); mark.busman@usda.gov (M.B.); martha.vaughan@usda.gov (M.M.V.); 2 Department of Agronomy & Plant Genetics, University of Minnesota, St. Paul, MN 55108, USA; ander319@umn.edu (J.A.A.); garvi007@umn.edu (D.F.G.)

**Keywords:** wheat, fusarium head blight, climate resilience, *Fhb1*, elevated CO_2_, mycotoxins, food safety

## Abstract

Fusarium head blight (FHB) is a destructive fungal disease of wheat that causes significant economic loss due to lower yields and the contamination of grain with fungal toxins (mycotoxins), particularly deoxynivalenol (DON). FHB disease spread and mycotoxin contamination has been shown to worsen at elevated CO_2_, therefore, it is important to identify climate-resilient FHB resistance. This work evaluates whether wheat with the *Fhb1* quantitative trait locus (QTL), the most widely deployed FHB resistance locus in wheat breeding programs, provides reliable disease resistance at elevated CO_2_. Near-isogenic wheat lines (NILs) derived from either a highly FHB susceptible or a more FHB resistant genetic background, with or without the *Fhb1* QTL, were grown in growth chambers at ambient (400 ppm) and elevated (1000 ppm) CO_2_ conditions. Wheat was inoculated with *Fusarium graminearum* and evaluated for FHB severity. At elevated CO_2_, the NILs derived from more FHB-resistant wheat had increased disease spread, greater pathogen biomass and mycotoxin contamination, and lower rates of DON detoxification; this was not observed in wheat from a FHB susceptible genetic background. The *Fhb1* QTL was not associated with increased disease severity in wheat grown at elevated CO_2_ and provided reliable disease resistance.

## 1. Introduction

Fusarium head blight (FHB) is a pervasive fungal disease of cereal crops which causes significant economic damage [1,2]. In north America, the primary causal agents of the disease are members of the *Fusarium graminearum* species complex [3,4]. Fungal spores initially infiltrate through exposed florets and then hyphae quickly colonize the wheat head through the rachis [5]. As the infection advances, *F. graminearum* produces deoxynivalenol (DON), a mycotoxin that causes significant plant cell death and leaves grains withered and contaminated [1,6]. Yield and quality suffer significantly and the harvested grain, contaminated with mycotoxins, may be unsuitable for human or animal consumption [7].

The likelihood and severity of FHB infection are highly dependent on environmental conditions and field management practices. Crop residues, particularly corn stover and wheat chaff, can significantly increase the likelihood of FHB infection by providing a refuge for the pathogen to overwinter and support the formation of fungal fruiting bodies, perithecia, as the environment becomes favorable [8,9]. Changes in climate are likely to shift pathogen populations and promote more severe and unpredictable disease outbreaks due to the intricate interplay between climatic, genetic, and agronomic factors [10,11,12,13]. Effective control of FHB often includes multiple integrated strategies including crop rotation, tillage/debris management, fungicide application, and the selection of FHB-resistant wheat varieties [14,15]. Of particular concern to breeders and growers is maintaining FHB resistance in wheat without compromising grain yield and quality; under-performing resistant varieties are likely to be abandoned [9]. Furthermore, recent studies have shown that the severity of FHB infection is expected to increase with rising atmospheric carbon dioxide (CO_2_) concentrations, prompting a search for disease-resistant cultivars with climate resilience [16,17].

Resistance to FHB is often categorized by the trait effect: Type I entails resistance to initial infection, Type II is resistance to fungal spread, and Type V is the de-toxification of mycotoxins, e.g., glycosylation of DON to DON-3-glucoside, by the host [18,19,20]. However, there is currently no known trait or combination of traits which provide complete resistance to FHB. There are multiple quantitative trait loci (QTL) that provide a stable degree of resistance to the disease: *Fhb1*, *Fhb2*, *Fhb5*, and *Fhb7*; with *Fhb1* being the most widely deployed. The *Fhb1* QTL was first identified from the Sumai 3 wheat cultivar on the short arm of the 3B chromosome [21,22,23]. Nearly all FHB resistance in north America in hard red spring wheat is derived from Sumai 3, with most breeding efforts focused on the incorporation of the *Fhb1* QTL [24]. While *Fhb1* helps slow the spread of the pathogen, it is not involved in the formation of D3G; however, the ability to detoxify DON has also been mapped near *Fhb1* on the short arm of chromosome 3B [25,26]. 

Functional validation of *Fhb1* has not been resolved [26,27,28,29,30]. Within the *Fhb1* locus, a pore-forming toxin-like gene (*PFT*) was identified and found to confer FHB resistance [26]. This gene was predicted to encode for a chimeric lectin that may disrupt the cell membrane of fungal pathogens. However, the *PFT* gene was also found in FHB-susceptible wheat and may be associated with general plant defense responses to biotic and abiotic stresses [27]. FHB resistance from *Fhb1* may also be due to a deletion mutation in *TaHRC*, a histidine-rich calcium-binding-protein gene [31,32]. Another candidate gene recently identified in the *Fhb1* locus, *TaLAC4*, is associated with increased FHB resistance through the lignification and thickening of secondary cell walls. This gene was predicted to encode a laccase protein that catalyzes lignin polymerization in the wheat rachis [28]. Furthermore, *TaNAC032*, a transcription factor within the Fhb1 locus, regulates *TaLAC4* and other lignin biosynthesis genes [29]. Resistance to FHB in the Sumai 3 cultivar is likely due to several general plant defense mechanisms: the induction of phenylpropanoids, the accumulation of lignin precursors, and the deposition of antifungal/antioxidant flavonoids at the cell wall all contribute to the reduction in pathogen spread and DON contamination [33].

The Sumai 3 cultivar has multiple major and minor QTLs associated with FHB resistance and extensive breeding efforts have been conducted to incorporate these resistance factors into elite wheat breeding lines; however, producing lines with acceptable crop performance has been exceedingly difficult [34,35,36]. Concerningly, recent studies with 26 wheat lines found that increased FHB resistance was correlated with decreased grain protein content at elevated atmospheric CO_2_ [37,38]. However, *Fhb1* was not associated with the disproportionate loss in grain protein content [38]. Additionally, wheat grown at elevated CO_2_ was found to be more susceptible to FHB spread though this effect was plant cultivar and pathogen strain specific [17]. 

Since the *Fhb1* QTL is widely utilized for FHB Type II resistance in wheat breeding and wheat has been observed to become more susceptible to FHB spread with rising CO_2_, it is vitally important to determine whether wheat with *Fhb1* retain disease resistance efficacy at elevated CO_2_. While changes in temperature and precipitation will affect the likelihood of FHB infection based on geographic location, the rising atmospheric CO_2_ concentration will impact all crops globally. It is crucial for breeders to know if key loci, targeted for disease resistance, will remain reliable under future atmospheric CO_2_ concentrations. 

In this study, near-isogenic wheat lines (NIL) derived from either a highly FHB susceptible or a more resistant genetic background, with or without the *Fhb1* QTL (Table 1), were grown in a completely random block design at ambient (400 ppm) and elevated (1000 ppm) CO_2_ conditions. The near-isogenic lines were inoculated with *Fusarium graminearum* and evaluated for disease progression, mycotoxin contamination, and fungal biomass accumulation. Differences in FHB disease severity were analyzed to determine if the *Fhb1* QTL provided reliable FHB resistance at elevated CO_2_. Furthermore, FHB severity was evaluated to determine whether the wheat genetic background altered *Fhb1* disease resistance performance, particularly at elevated CO_2_. Furthermore, since a number of glycosyltransferases are known to be located near the *Fhb1* QTL region on chromosome 3B and the fact that this proximity has led to speculation that *Fhb1* was associated with DON detoxification, we also looked at the proportion of DON to D3G conversion in the NIL lines at ambient and elevated CO_2_.

## 2. Results

**Figure 1:**
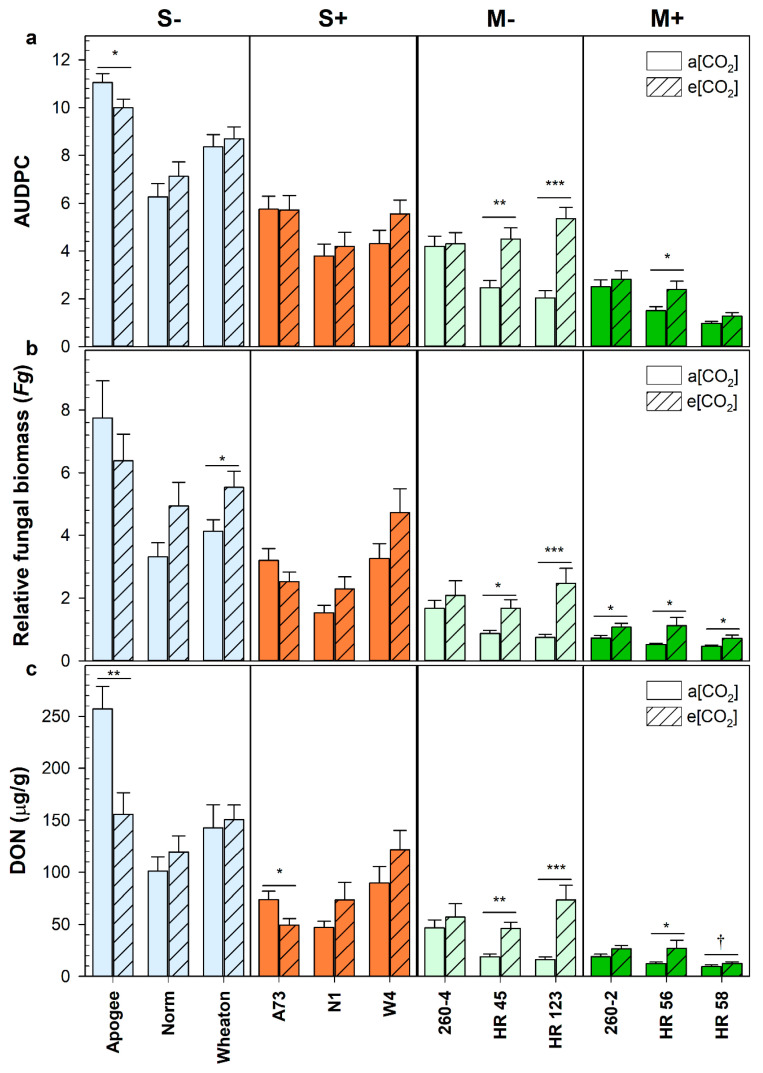
Disease severity of *Fusarium graminearum* infected wheat heads as defined by(**a**) the area under the disease progression curve (AUDPC), (**b**) deoxynivalenol (DON) contamination, and (**c**) relative fungal biomass (*Fg*). Genotypes were derived from either a susceptible check (S) or a more resistant genetic background (M) and each either possessed the *Fhb1* QTL, as indicated by a (+), or lacked the *Fhb1* QTL, as indicated by a (−). Wheat was grown at either ambient (a[CO_2_]) or elevated carbon dioxide concentrations (e[CO_2_]). Error bars represent the standard error. Symbols (†, *, **, and ***) denote a statistically significant effect (*p* < 0.1, *p* < 0.05, *p* < 0.01, and *p* < 0.0001, respectively) of elevated CO_2_ on AUDPC, DON, or *Fg* within a genotype, as determined by a non-parametric Van der Waerden test (AUDPC: *n* = 45 and DON and *Fg*: *n* = 15).

The *Fhb1* QTL significantly reduced disease severity in wheat, maintaining a consistent percent reduction in disease spread and mycotoxin contamination regardless of genetic background or atmospheric CO_2_ (Figure 1). However, the genetic background of the wheat genotypes significantly impacted disease severity at elevated CO_2_, compared with ambient levels. Genotypes HR45, HR123, and HR56, derived from a more resistant genetic background (M), had significantly greater disease severity when grown at elevated CO_2_; the genotypes displayed greater disease spread (Figure 1a), greater relative fungal biomass accumulation (Figure 1b), and greater DON contamination (Figure 1c). 

**Figure 2:**
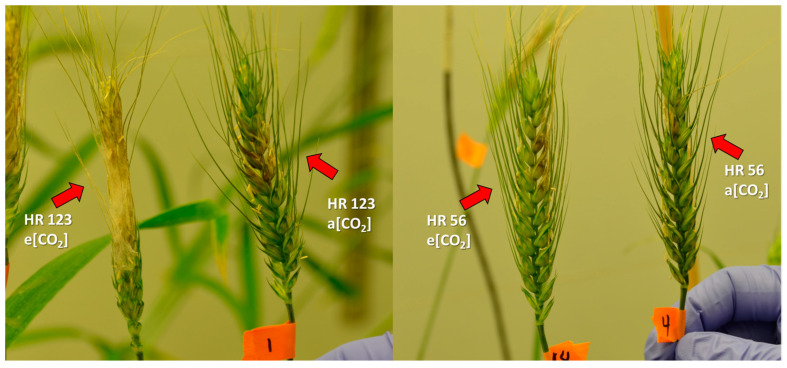
Wheat genotypes HR123 (M−) and HR 56 (M+) at one week post single floret inoculation of *Fusarium graminearum*. Both cultivars exhibit more severe FHB disease spread at elevated CO_2_.

Increased pathogen biomass was observed in all but one M genotype at elevated CO_2_. The increased disease severity was already apparent only one week after point inoculation, with more rapid disease spread in the M genotypes at elevated CO_2_ (Figure 2). Increased disease severity at elevated CO_2_ was most pronounced in the HR123 genotype, which had severely accelerated disease spread and a more than five-fold increase in DON accumulation (Figure 1a,c). Not all M genotypes were so severely affected as neither 260-4 nor 260-2 had a significant increase in toxin accumulation at elevated CO_2_. However, both genotypes had relatively poor FHB resistance at ambient CO_2_, with 2- to 4-fold greater mycotoxin accumulation compared to the other genotypes within their *Fhb1* group (M− or M+, respectively).

In comparison, the susceptible genotypes (S) had no increased susceptibility to FHB when grown at elevated CO_2_, with the sole exception of Wheaton, which had an increase in relative fungal biomass (Figure 1b). Surprisingly, the susceptible genotype Apogee, as well as its NIL A73 (*Fhb1*+), had less disease spread and DON accumulation at elevated CO_2_ when compared with plants grown at ambient CO_2_ (Figure 1a,c). However, while Apogee had reduced disease spread and DON accumulation at elevated CO_2_, it had the highest DON contamination, disease spread, and fungal biomass accumulation of any genotype tested at either ambient or elevated CO_2_.

**Figure 3:**
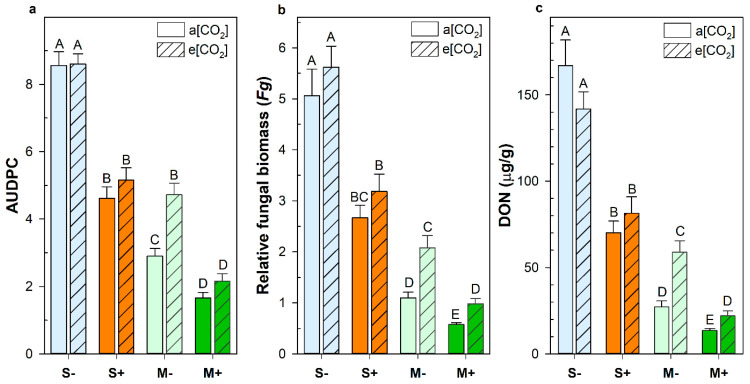
Disease severity in various wheat groups, as defined by (**a**) the area under the disease progression curve (AUDPC), (**b**) the relative fungal biomass in infected wheat heads (*Fg*), and (**c**) the accumulation of deoxynivalenol (DON) in wheat heads at either ambient (a[CO_2_]) or elevated carbon dioxide concentrations (e[CO_2_]). Error bars represent standard error. Different letters denote statistically significant differences as determined by a non-parametric Wilcoxon/Kruskal–Wallis for multiple pairwise comparisons (AUDPC: *n* = 135; DON and *Fg*: *n* = 45).

When analyzing differences between wheat groups, S genotypes were not impacted by atmospheric CO_2_ but the M genotypes had more severe FHB infections when grown at elevated CO_2_ (Figure 3). The M- genotypes that lacked the *Fhb1* QTL had 63% more disease spread (*p* < 0.0001), 89% more fungal biomass (*p* = 0.0003), and 109% more DON accumulation (*p* < 0.0001) at elevated CO_2_ compared to ambient. While the M+ genotypes had less overall disease, due to the presence of the *Fhb1* QTL, they had 70% more fungal biomass (*p* < 0.0001) and 52% more DON (*p* = 0.0065) at elevated CO_2_ (Figure 3). The *Fhb1* QTL provided significant FHB disease resistance regardless of the wheat genetic background or CO_2_ condition. On average, the *Fhb1* QTL reduced DON by 47% (*p* < 0.0001), disease spread or area under the disease progression curve (AUDPC) by 45% (*p* < 0.0001), and pathogen fungal biomass (*Fg*) by 46% (*p* < 0.0001). While the *Fhb*1 QTL reduced disease severity in wheat, the genetic background had a greater impact on disease outcome; the M genotypes had 57% less disease spread (*p* < 0.0001), 71% less fungal biomass (*p* < 0.0001), and 77% less DON (*p* < 0.0001) compared to susceptible cultivars. Furthermore, in a permutational multivariate analysis of variance to determine what factors contributed most to the variance in disease severity, wheat genetic background accounted for 34%, while *Fhb1* accounted for only 10% (Table S1). Even without the *Fhb1* QTL, the M− genotypes maintained a substantial degree of resistance against FHB disease spread and toxin contamination (Table S2 and Figure 3). Only at elevated CO_2_ was disease spread comparable between S+ and M− wheat (*p* = 0.3791); however, both DON and fungal biomass accumulation remained significantly lower in M− genotypes (*p* = 0.0005 and *p* = 0.0058, respectively).

**Figure 4:**
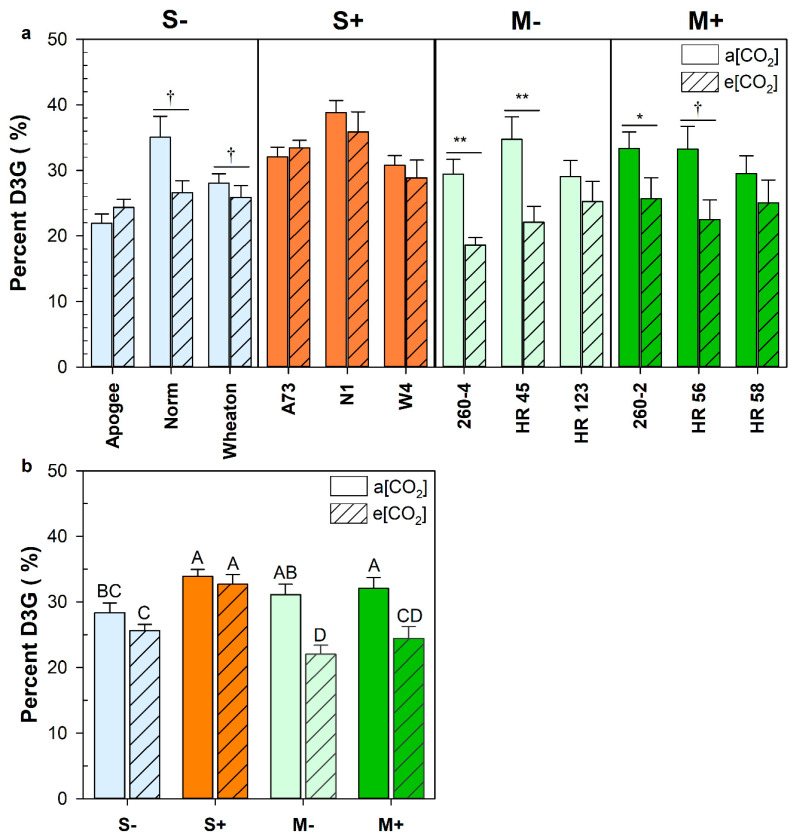
The percentage of deoxynivalenol-3-glucoside (D3G), on a molar basis, in total measured trichothecenes in wheat cultivars (**a**) and by group (**b**) at either ambient (a[CO_2_]), or elevated carbon dioxide concentrations (e[CO_2_]). Error bars represent standard error. Symbols (†, *, **) denote a statistically significant effect of elevated CO_2_ on disease characteristics within a group (*p* < 0.1, *p* < 0.05; respectively), as determined by a non-parametric Van der Waerden test ((**a**): *n* = 15). Different letters denote statistically significant differences as determined by a non-parametric Wilcoxon/Kruskal–Wallis for multiple pairwise comparisons ((**b**): *n* = 45).

The percent of deoxynivalenol-3-glucoside (D3G) was evaluated to determine whether differences in glycosylation rate could account for altered FHB severity at elevated CO_2_. The proportion of DON to D3G was significantly less for most of the M genotypes grown at elevated CO_2_ (Figure 4a). The decrease in percent D3G was associated with the M genetic background but was not associated with the *Fhb1* QTL (Figure 4b). Interestingly, Apogee (*Fhb1−*) and A73 (*Fhb1+*) differed significantly in percent D3G (*p* < 0.0001), despite being derived from the same parental genetic background. 

**Figure 5:**
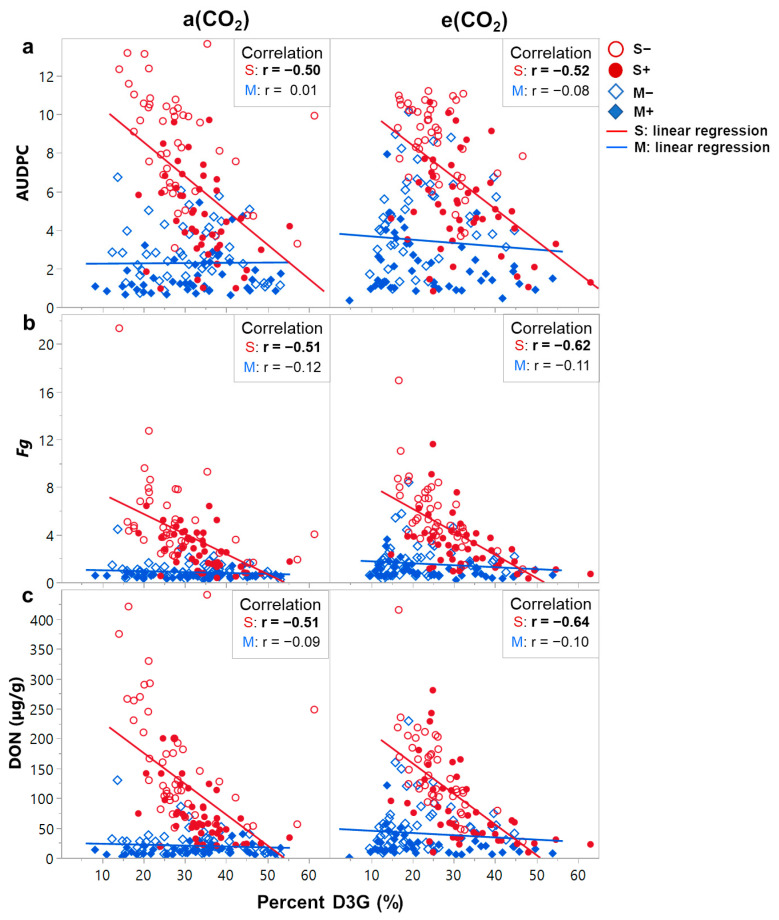
Correlations between the percentage of deoxynivalenol-3-glucoside (D3G) and FHB disease metrics: (**a**) area under the disease progression curve (AUDPC), (**b**) relative *Fusarium graminearum* biomass (*Fg*), and (**c**) deoxynivalenol (DON) contamination at either ambient (a[CO_2_]) or elevated carbon dioxide concentrations (e[CO_2_]). Significant correlations are highlighted in bold text (α = 0.05).

While reduced plant detoxification of DON into D3G could account for some of the increased disease severity, percent D3G was poorly correlated with disease severity metrics in the M genotypes (Figure 5). Only in the susceptible wheat genotypes was the percentage of DON to D3G negatively correlated with disease severity. In the M genotypes, percent D3G had no significant correlation to disease spread, fungal biomass, or DON contamination. Therefore, changes in DON glycosylation, while significantly reduced in M genotypes at elevated CO_2_, was not the principal cause of increased FHB severity.

## 3. Discussion

Our results demonstrate that the *Fhb1* QTL provided reliable FHB resistance in wheat, even at elevated CO_2_. Furthermore, *Fhb1* provided a consistent percent reduction in disease severity regardless of the wheat genetic background: 45–47% reduction in disease progression, DON contamination, and pathogen biomass. This was consistent with previously reported FHB disease resistance provided by the *Fhb1* region in the Sumai 3/Stoa NIL lines [23]. However, the overall disease severity was primarily dependent on the inherent FHB resistance, or lack thereof, in the wheat genetic background. While *Fhb1* reduced FHB spread in S+ genotypes compared with the highly susceptible S− genotypes, mycotoxin contamination was still substantial in all wheat from a susceptible genetic background. 

M genotypes from the Sumai 3/Stoa RIL 63–4//MN97448 pedigree suffered increased disease susceptibility to FHB spread at elevated CO_2_ unlike wheat from susceptible genetic backgrounds. However, the *Fhb1* QTL was not associated with increased relative disease susceptibility at elevated CO_2_ as M- genotypes, which lacked *Fhb1*, had more severe FHB infections and contamination when grown at elevated CO_2_. FHB disease resistance in wheat has been associated with the loss of grain protein and mineral content at elevated CO_2_ [37,38]. The accessibility of host nutrients and the availability of nitrogen can substantially influence the expression of *F. graminearum* genes associated with virulence and pathogenicity [39,40]. Fungal pathogens often have a shared promotor region that links virulence-associated genes with major nitrogen regulatory transcription factors [41]. The *F. graminearum* strain used in this study (9F1) has been previously observed to increase mycotoxin biosynthesis in response to the loss of wheat nutritional content [42]. Furthermore, the 9F1 strain has caused differential disease severity in wheat depending on the CO_2_ concentration and the genetic background of the host, though that study employed a whole-head inoculation method rather than the single floret inoculation method used in this study [17]. As we observed in the M genotypes, *F. graminearum* had greater disease spread, greater fungal biomass, and more DON accumulation in the moderately resistant wheat cultivar Alsen—harboring *Fhb1* and derived from Sumai 3—when it was grown at elevated CO_2_.

In the current study, the most significant contributing factor to overall disease resistance was the wheat genetic background. Plant defense against pathogens can involve physiological, phytochemical, and compositional adaptations as well as a plethora of kinases, glycosyltransferases, antimicrobial peptides, and apoplastic proteases, though these defenses are often countered by antagonistic microbial virulence factors [18,43,44,45]. While the *Fhb1* QTL from Sumai 3 is the most widely studied and deployed, there are numerous other wheat QTLs associated with FHB resistance; most are considered minor contributors to plant defense but few have been validated and incorporated into breeding programs [46,47]. However, the stacking or combination of multiple resistance alleles can significantly enhance wheat defenses against head blight [48]. This is evidenced in our results (Figure 3) where the M− genotypes still had strong disease resistance even though the expected main source of resistance, *Fhb1*, was bred out of the NILs. Combining the highly effective *Fhb1* QTL with the putative resistance traits within the M genetic background resulted in the lowest disease severity, even with worsening FHB infection and DON contamination at elevated CO_2_. We did find a significant Fhb1*genetic background interaction (*p* < 0.0001; Table S1), indicating that *Fhb1* was more effective in more resistant genetic backgrounds. This suggests synergistic interactions between *Fhb1* and the yet unidentified resistance factors within the M genotypes. However, fully elucidating these interactions is beyond the scope of the current work.

Identifying specific resistance genes and evaluating their functionality is an incredibly arduous undertaking. Furthermore, unwanted or deleterious genes often accompany the desired resistance trait during plant breeding, resulting in linkage drag or the reduction in plant fitness from the genetic stowaways [36]. While *Fhb1* is not associated with an altered plant response or increased FHB susceptibility at elevated CO_2_, the M genetic background harbors currently unidentified detrimental trait/s that cause these effects. From our results, we found a consistent reduction in type II and type V resistance in M genotypes at elevated CO_2_. 

The *Fhb1* QTL only provides Type II resistance or the inhibition of disease spread and severity and does not affect the likelihood of infection [21,23]. Wheat is also capable of converting the phytotoxic DON into D3G, detoxifying the trichothecene through the addition of glucose—a form of type V resistance [49]. The modification of bioactive compounds by glycosylation, namely the conjugation of a sugar molecule to a compound via a glycosyltransferase enzyme, is a common feature of plant defense used to alter toxin bioavailability to hosts or pathogens [50,51]. The wheat genome contains hundreds of glycosyltransferases located among all chromosomes and across all three sub-genomes (A, B, and D) that strongly respond to pathogen infection. In one study, 59% of these glycosyltransferases were upregulated two days after infection by a DON-producing *F. graminearum* strain compared with control wheat [52]. However, the ability of wheat to convert DON to D3G was only helpful in reducing disease severity for the susceptible genotypes where the percentage of DON converted was negatively correlated with disease spread, fungal biomass, and total DON accumulation (Figure 5). We had hypothesized that the glycosylation of DON and, therefore, the inactivation of its phytotoxicity, would be an important factor in plant FHB resistance but the conversion of DON to D3G appears to provide minimal protection compared to other resistance factors found within the Sumai 3 genetic background. Our results suggest that it may be advantageous in terms of FHB resistance to seek wheat breeding strategies that incorporate non-native or novel DON detoxifying mechanisms—such as *Fhb7* [53]—that could provide more protection than native wheat glycosyltransferases.

The waning FHB resistance of M genotypes with rising CO_2_ is concerning for the future of FHB mitigation in wheat. Utilizing FHB-resistant wheat cultivars is an essential component in an effective integrated management strategy to reduce FHB damage [54]. Further breeding efforts are needed to avoid diminishing FHB resistance, coupled with reduced grain nutritional quality, as atmospheric CO_2_ concentrations increase. But despite worsening disease severity in the more FHB-resistant wheat, they remained the most resilient to mycotoxin contamination with rising CO_2_ (Figure 3). Therefore, the use of FHB resistance factors from Sumai 3, particularly *Fhb1*, should still be utilized in wheat breeding programs for reducing mycotoxin contamination and disease damage. Efforts are ongoing to identify the cause of the detrimental CO_2_ response in the M genotypes. It is vitally important to identify climate-resilient and disease-resistant wheat cultivars to ensure future food safety and security.

## 4. Materials and Methods

### 4.1. Fhb1 Near-Isogenic Lines

Two sets of NILs with varying inherent FHB resistance were used to determine the impact of elevated CO_2_ on *Fhb1* disease resistance efficacy. Details on the backcrossing and characterization of the susceptible NILs were reported previously [38]. Briefly, the first set of NILs were produced from highly FHB susceptible cultivars (S), Norm [55], Wheaton [56], and Apogee [57,58]. Apogee, Norm, and Wheaton are often used as susceptible checks in FHB research. For this manuscript, genotypes derived from these highly FHB susceptible cultivars are designated as genetic background ‘S’. Sumai 3 was used as the donor of *Fhb1* to generate near-isogenic lines in the three susceptible genotypes using marker-assisted backcrossing. The *Xgwm493* molecular marker [59] was used to select for *Fhb1* in four generations with each respective genotype serving as a recurrent parent. The susceptible BC_4_F_3_ *Fhb1* near-isogenic lines are designated N1 (Norm near-isogenic line), W4 (Wheaton near-isogenic line, and A73 (Apogee near-isogenic line) and are more than 97% homozygous with the recurrent parent genome. NIL genotypes from the susceptible check genetic background are referred to as S− (for those without the *Fhb1* QTL) and S+ (for those with the *Fhb1* QTL). 

The second set of NILs was developed using a moderately susceptible spring wheat background that has been used for the fine mapping of *Fhb1* [60]. The choice of the FHB moderately susceptible background was to allow for the characterization of genotypes that lacked the FHB resistance provided by *Fhb1*, i.e., mapping the genomic region harboring *Fhb1*. For the purpose of clarity, this group of NILs is designated genetic background ‘M’. Genotypes that possess a functional *Fhb1* QTL were classified as moderately resistant (M+) while those without *Fhb1* were classified as moderately susceptible (M−). The NIL set was derived from a single F7 plant that was heterozygous for *Fhb1* and all NILs have the pedigree Sumai 3/Stoa RIL 63–4//MN97448. The genotypes are designated 260-2, HR 45, HR 56, HR 58, HR 123, and 260-4. All NIL lines were examined previously for agronomic performance at elevated CO_2_ [38].

### 4.2. Wheat Growing Conditions

To evaluate whether FHB resistance from the *Fhb1* QTL is diminished at elevated CO_2_, the NIL wheat genotypes (Table 1) were grown in PGR15 environmentally controlled growth chambers capable of CO_2_ fumigation (Controlled Environments INC., Winnipeg, MB, Canada). Growing conditions were similar to those reported previously [38]. Briefly, plants were grown in six chambers, three set to ambient CO_2_ (420 ± 10 ppm, a[CO_2_]) and three set to elevated CO_2_ (1000 ± 10 ppm [CO_2_], (e[CO_2_]). The elevated CO_2_ treatment was chosen based on the predicted atmospheric CO_2_ concentration at the end of the century in the highest emissions scenario (SSPS-8.5) [61]. For each chamber, wheat genotypes were grown in triplicate 20 × 15-cm plastic pots filled with approximately 4 L of SunGrow Horticulture potting mix (Agawam, MA, USA), with 5 plants per pot. The experiment was performed twice, evaluating half of the NILs at a time. The wheat was grown at 25/23 °C (day/night) and 50–60% relative humidity. The chamber light source was from a mix of incandescent and fluorescent lights set to a 14 h photoperiod (550 μmol m^−2^ s^−1^ photosynthetic photon flux density). Plants were well watered, typically daily, and received additional biweekly fertilization with Peters 20-20-20 nutrient supplement (The Scotts Company, Marysville, OH, USA) until anthesis. 

### 4.3. Disease Assays

To evaluate *Fhb1* disease resilience at elevated CO_2_, the *F. graminearum* isolate 9F1 (NRRL 37676) was used to inoculate wheat cultivars (Table 1) at anthesis (flowering). The 9F1 strain is a DON producer originally isolated from wheat in the Netherlands. The preparation of media, pathogen culturing, and wheat inoculations were performed according to previously reported methods [42]. Briefly, fungal isolates were grown on V8 agar plates for 7 d before an agar plug was transferred into 20 mL of sterile mung bean broth. Cultures were grown for 48 h, at 28 °C, and 160 rpm under dark conditions in a New Brunswick Innova 44 incubator shaker (Eppendorf, Hauppauge, NY, USA) to promote conidia formation. The culture was centrifuged and the mung bean supernatant was discarded. Afterwards, the conidia pellet was resuspended in 0.04% Tween 20 (Thermo Fisher Scientific, Waltham, MA, USA) to form a 1 × 10^5^ mL^−1^ conidia suspension. The suspension was used for wheat floret inoculations immediately after preparation.

Fifteen wheat heads per line, per chamber, were tagged and inoculated at flowering with 10 µL of the conidial suspension into single florets. Immediately after inoculation, plastic bags were placed over the wheat heads to maintain a high humidity environment. The plastic bags were supported by a central bamboo stake to prevent lodging or severe stem bending and the stake(s) and bag(s) were removed after 3 d. Disease progression and the area under the disease progression curve (AUDPC) was determined by visual assessment of the number of diseased florets (identified as bleached or necrotic plant tissues) on days 7, 10, 14, 17, and 21 after inoculation. At day 21, the infected wheat heads were collected and stored at −80 °C before being lyophilized and ground for mycotoxin and pathogen biomass analysis.

### 4.4. Mycotoxin Analyses

To determine how *Fhb1* impacted mycotoxin accumulation in wheat heads at ambient and elevated CO_2_, we measured both deoxynivalenol (DON) and DON-3-glucoside (D3G) in infected wheat heads collected 21d after inoculation. Briefly, 0.5 g of ground infected wheat heads was extracted with 10 mL of acetonitrile/water (86:14, vol/vol). An aliquot of the extract was analyzed for glycosylated deoxynivalenol (deoxynivalenol-3-glucoside; D3G) content using liquid chromatography/mass spectrometry coupled with electrospray ionization tandem mass spectroscopy (LC-ESI-MS/MS) on an Agilent 1100 HPLC (Agilent Technologies, Inc., San Clara, CA, USA) linked with a SCIEX 3500 TripleQuad MS (AB SCIEX LLC, Framingham, MA USA). Samples were separated on a Phenomenex (Torrance, CA, USA) Kinetex C18 column (150 mm length, 4.6 mm diameter, 2.6 μm particle size) following a previously reported methodology [19]. A separate 5 mL aliquot of each acetonitrile/water extract was cleaned with a MycoSep 225 column (Romer Labs, Union, MO, USA). To prepare DON trimethylsilyl (TMS) derivatives, a 2 mL aliquot of the purified extract was dried and reacted with 100 μL of a 100:1 mixture of N-methylsilylimadazole/trimethylchlorosilane (Sigma-Aldrich, St. Louis, MO, USA). After 30 min, 1 mL of water and 900 μL isooctane were added and the mixture was gently vortexed. The top isooctane fraction was analyzed with GC-MS on an Agilent 7890 gas chromatograph (Agilent Technologies, Santa Clara, CA, USA) fitted with a HP-5MS column (30 m, 0.25 mm, 0.25 μm) and a 5977 mass detector using selective ion monitoring. TMS derivatives of purified DON (0.3125 to 80 μg) were similarly prepared to construct a standard curve.

### 4.5. Estimation of Host and Pathogen Biomass

The assessment of relative fungal biomass in diseased wheat heads was determined using the ratio of *Fg* DNA to wheat DNA via quantitative polymerase chain reaction (qPCR) according to the methods reported previously [42]. Three technical replications were performed per sample. The primers and probes used for qPCR can be found in Table 2. The approximate fungal biomass (relative *Fg* DNA/wheat DNA) was calculated by dividing the geometric mean of initial DNA concentration (N_0_) from the *Fusarium* probes by the geometric mean of N_0_ from the wheat probes. Finally, the amount of DON production per unit biomass was estimated by dividing the µg g^−1^ DON by the relative pathogen biomass, as quantified by qPCR.

### 4.6. Statistical Analyses

Results were evaluated using a non-parametric Van der Waerden test (α = 0.05) to determine significant differences due to elevated CO_2_. Subsequently, a post hoc analysis was performed evaluating group differences via a multiple pairwise comparison using a non-parametric Wilcoxon/Kruskal–Wallis test (α = 0.05). Pearson correlations between independent variables were determined by a multivariate analysis, α = 0.05 (JMP V15.0). A principal component analysis was performed in JMP V15.0. Additionally, a permutational multivariate analysis of variance was performed in R 4.2.1 (adonis2; vegan package) to analyze sources of variation in disease severity treatments. Details on pairwise comparisons can be found within the table and figure legends.

## 5. Conclusions

The *Fhb1* QTL was not associated with increased disease susceptibility at elevated CO_2_ and provided reliable FHB resistance. However, wheat from the M genetic background suffered greater FHB disease progression, DON contamination, and pathogen biomass accumulation at elevated CO_2_. Furthermore, there was a reduction in the detoxification of DON into D3G but this alone did not account for the increased disease severity. The wheat NILs derived from susceptible genetic backgrounds did not have greater disease severity or altered rates of DON detoxification at elevated CO_2_. Inserting *Fhb1* into susceptible cultivars can significantly reduce disease, but alone, the QTL does not provide robust protection; a combination of resistance factors will likely be required to strongly suppress FHB infection. The use of FHB resistant cultivars and traits, including those derived from Sumai 3, will likely remain one of the most effective strategies to reduce disease and mycotoxin contamination in wheat. However, it is essential to develop climate resilient disease resistant wheat to ensure global food safety and security.

## Figures and Tables

**Table 1:** Breeding pedigrees for wheat cultivars in the current study. Wheat genotypes are either derived from backcrossing *Fhb1* from Sumai 3 into susceptible wheat cultivars (S background) or by identification of near-isogenic lines (NILs) in a moderately susceptible (M) background. Lines containing the *Fhb1* QTL are indicated with a “+” and those without *Fhb1* with a “−”.

Genotype	Genetic Background	*Fhb1* QTL	Group	Pedigree
Apogee	S	−	S−	Apogee
Norm	S	−	S−	Norm
Wheaton	S	−	S−	Wheaton
A73	S	+	S+	Apogee*5/Sumai 3
N1	S	+	S+	Norm*5/Sumai 3
W4	S	+	S+	Wheaton*5/Sumai 3
260-4	M	−	M−	Sumai 3/Stoa RIL 63–4//MN97448
HR 45	M	−	M−	Sumai 3/Stoa RIL 63–4//MN97448
HR 123	M	−	M−	Sumai 3/Stoa RIL 63–4//MN97448
260-2	M	+	M+	Sumai 3/Stoa RIL 63–4//MN97448
HR 56	M	+	M+	Sumai 3/Stoa RIL 63–4//MN97448
HR 58	M	+	M+	Sumai 3/Stoa RIL 63–4//MN97448

**Table 2:** Primer and probe sequences used for quantitative polymerase chain reaction (qPCR) amplification. Three sets of primers and matching probes were used to quantify relative fungal or wheat biomass.

Primer Name	Organism	Gene Product	Primer Sequence
Fg.Tri101 Forward	*F. graminearum*	Trichothecene 3-*O*-acetyltransferase	GGACTCTGGGATTACGACTTTG
Fg.Tri101 Reverse	*F. graminearum*	Trichothecene 3-*O*-acetyltransferase	ATCAGGCTTCTTGGGCATAAA
Fg.Tri101 Probe	*F. graminearum*	Trichothecene 3-*O*-acetyltransferase	CGAGACTGTGAGACGGCCAATCTTT
Fg.TEF Forward	*F. graminearum*	Translation elongation factor	CAGTCACTAACCACCTGTCAAT
Fg.TEF Reverse	*F. graminearum*	Translation elongation factor	AATGGTGATACCACGCTCAC
Fg.TEF Probe	*F. graminearum*	Translation elongation factor	AACCCAGGCGTACTTGAAGGAACC
Fg.RED Forward	*F. graminearum*	Reductase	TGACAGCTTTGGTTGTGTTTG
Fg.RED Reverse	*F. graminearum*	Reductase	CTTGGCTGGAATGAGTCTGT
Fg.RED Probe	*F. graminearum*	Reductase	CGGAAGACTGCTGAGTAACGCCAA
Ta.Ef1 Forward	*T. aestivum*	Elongation factor	GATTGACAGGCGATCTGGTAAG
Ta.Ef1 Reverse	*T. aestivum*	Elongation factor	GGCTTGGTGGGAATCATCTT
Ta.Ef1 Probe	*T. aestivum*	Elongation factor	TCCTCAAGAATGGTGATGCTGGCA
Ta.Actin Forward	*T. aestivum*	Actin	CCAAGGCCAACAGAGAGAAA
Ta.Actin Reverse	*T. aestivum*	Actin	GCTGGCATACAAGGACAGAA
Ta.Actin Probe	*T. aestivum*	Actin	TGCCCAGCAATGTATGTCGCAATC
Ta.PAL Forward	*T. aestivum*	Phenylalanine ammonia-lyase	GTGTTCTGCGAGGTGATGAA
Ta.PAL Reverse	*T. aestivum*	Phenylalanine ammonia-lyase	GTATGAGCTTCCCTCCAAGATG
Ta.PAL Probe	*T. aestivum*	Phenylalanine ammonia-lyase	AAGCACCACCCTGGACAGATTGAA

## Data Availability

The datasets generated during and/or analyzed during the current study are available from the corresponding author upon reasonable request.

## Supplementary Materials

The following supporting information can be downloaded at https://www.mdpi.com/article/10.3390/plants12203527/s1. Table S1: Permutational multivariate analysis of variance to analyze sources of variation in disease severity; Figure S1: Principal component analysis of FHB disease severity; Table S2: Average number of spikelets and the percent of the spikelets which exhibited FHB disease symptoms.
