# Supplementary material for: Elevated CO2 Can Worsen Fusarium Head Blight Disease Severity in Wheat but the Fhb1 QTL Provides Reliable Disease Resistance
Source: Plants (Basel). 2023 Oct 11;12(20):3527. doi: 10.3390/plants12203527 (PMC10610529; doi:10.3390/plants12203527)
Supplement: Supplementary file 1 [file plants-12-03527-s001.zip › plants-2649615-supplementary.pdf]

Supplementary Information:

Supplementary Table S1. Permutational multivariate analysis of variance to analyze sources of variation in disease severity (R 4.2.1). Analysis determines how plant genetic background, the *Fhb1* QTL, and CO_2 concentration explains variability in the statistical model. Symbols (†, ***) denote a statistically significant difference ($p < 0.1$, $p < 0.0001$; respectively).

	Df	Sum of squares	R^2	F	Pr (>F)	
Genetic Background	1	17.867	0.33656	239.518	0.001	***
Fhb1	1	5.063	0.09538	67.88	0.001	***
[CO_2]	1	0.875	0.01648	11.7296	0.001	***
Genetic Background: *Fhb1*	1	1.93	0.03635	25.8713	0.001	***
Genetic Background:[CO_2]	1	0.853	0.01606	11.4312	0.001	***
Fhb1:[CO_2]	1	0.177	0.00333	2.3706	0.102	
Genetic Background: *Fhb1*:[CO_2]	1	0.214	0.00403	2.8707	0.058	†
Residual	350	26.108	0.4918			
Total	357	53.087	1			

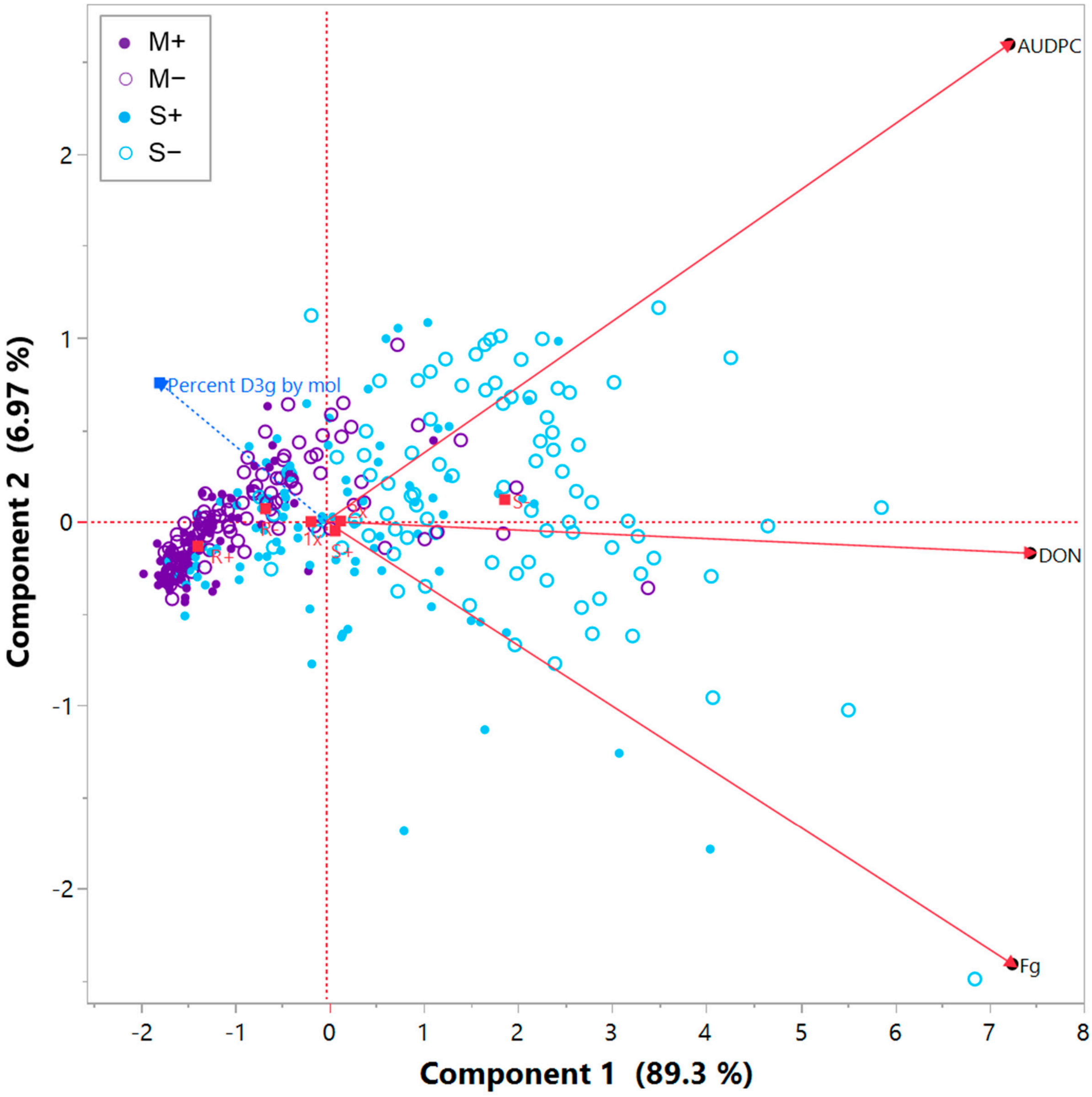

Supplementary Figure S1. Principal component analysis (PCA) of FHB disease severity – as defined as disease progression, fungal biomass, and toxin accumulation – in wheat cultivars grown at ambient and elevated CO_2. Arrows represent the influence of each independent variable on the principal components. The blue dished line did not influence the principal components, but only represents how percent D3G relates to the disease severity in this analysis.

Supplementary Table S2. Average number of spikelets and the percent of the spikelets which exhibited FHB disease symptoms 21 days after inoculation at ambient (a[CO_2]) and elevated (e[CO_2]) carbon dioxide concentrations. Error represents the standard error of the mean (n = 45).

Genotype	Group	Spikelets: a[CO_2]	Spikelets: e[CO_2]	% Disease: a[CO_2]	% Disease: e[CO_2]
Apogee	S−	15.89 ±0.13	16.93 ±0.12	93.70 ±2.65	92.61 ±2.52
Norm	S−	20.13 ±0.17	20.40 ±0.18	71.64 ±5.09	75.48 ±5.35
Wheaton	S−	19.71 ±0.13	20.69 ±0.18	87.95 ±3.88	89.37 ±3.57
A73	S+	15.56 ±0.12	16.38 ±0.11	57.26 ±5.09	53.71 ±5.46
N1	S+	20.24 ±0.19	20.64 ±0.20	41.54 ±5.84	48.00 ±6.12
W4	S+	20.44 ±0.14	21.64 ±0.17	48.32 ±6.17	62.20 ±6.13
260-4	M−	20.02 ±0.15	20.53 ±0.16	47.06 ±5.08	44.78 ±4.35
HR 45	M−	19.64 ±0.14	20.53 ±0.19	28.03 ±3.98	56.31 ±5.11
HR 123	M−	20.38 ±0.14	20.36 ±0.14	21.49 ±3.75	58.30 ±3.96
260-2	M+	20.13 ±0.19	21.47 ±0.17	24.18 ±3.30	25.05 ±3.71
HR 56	M+	19.84 ±0.14	20.84 ±0.17	12.36 ±2.09	16.61 ±2.28
HR 58	M+	19.49 ±0.17	20.67 ±0.15	6.78 ±0.52	8.07 ±0.94
